# Additions and corrections to the check list of the Noctuoidea (Insecta, Lepidoptera) of North America north of Mexico

**DOI:** 10.3897/zookeys.149.1805

**Published:** 2011-11-24

**Authors:** J. Donald Lafontaine, B. Christian Schmidt

**Affiliations:** 1 Canadian National Collection of Insects, Arachnids, and Nematodes, Biodiversity Program, Agriculture and Agri-Food Canada, K.W. Neatby Bldg., 960 Carling Ave., Ottawa, Ontario, Canada K1A 0C6; 2 Canadian Food Inspection Agency, Canadian National Collection of Insects, Arachnids, and Nematodes, K.W. Neatby Bldg., 960 Carling Ave., Ottawa, Ontario, Canada K1A 0C6

**Keywords:** Canada, United States, Notodontidae, Doidae, Erebidae, Euteliidae, Noctuidae, Nolidae, distribution, faunistics

## Abstract

A total of 115 additions and corrections are listed and discussed for the check list of the Noctuoidea of North America north of Mexico published in 2010. Thirty-two of these are changes in authorship and/or date of publication or spelling. Taxonomic changes are 33 new or revised synonymies, three new combinations, and six revisions in status from synonymy to valid species.

## Introduction

Continuing work on the check list of Noctuoidea of North America north of Mexico has resulted in 115 changes to the list published last year (Lafontaine and Schmidt 2010). About one-third of these (32) are corrections in date, authorship or spelling, but the majority is the result from continuing taxonomic work in comparing species and generic concepts with type material and examination of genital characters. Sixteen species are added to the fauna: two were omitted previously; six were described in the past year; five are newly reported from the United States, and three are former synonyms now recognized as valid species. Thirty species are deleted from the fauna through synonymy or re-identification, reducing the total number of species to 3679 (down from 3693).

## Materials and methods

### Repository abbreviations

Taxonomic changes are based on examination of material, especially type specimens, in the following collections:

BMNH The Natural History Museum [statutorialy: British Museum (Natural History)], London, UK

CNC Canadian National Collection of Insects, Arachnids, and Nematodes, Ottawa, Ontario, Canada

FMNH The Field Museum, Chicago, Illinois, USA

ODA Oregon Department of Agriculture, Salem, Oregon, USA

TLSRC Texas Lepidoptera Survey Research Collection, Houston, Texas, USA

USNM National Museum of Natural History [formerly, United States National Museum], Washington, District of Columbia, USA

ZMHB Museum für Naturkunde der Humboldt-Universität, Berlin, Germany

## Results

### Corrections, additions, and changes (highlighted in bold)

p. 2 & p. 14 Subfamily Lymantriinae Hampson

**Tribe Nygmiini Holloway, 1999** [after Tribe Leucomini]

p. 3 & p. 39 Subfamily Eulepidotinae Grote, **1895**

p. 3 & p. 39 Tribe Eulepidotini Grote, **1895**

p. 4 & p. 64 Subfamily Agaristinae **Boisduval, 1833**

930067 *Macrurocampa marthesia* (Cramer, **[**1780**]**)

930125 ***Elasmia****packardii* (Morrison, 1875)

930126 ***Elasmia cave* Metzler, 2011**

**syn. *Hippia insularis* of authors, not Grote, 1866**

**930126.1 *Elasmia mandela* (Druce, 1887)**

**ssp. *Elasmia mandela santaana* Metzler & Knudson, 2011**

930130 *Symmerista* ***suavis*** (Barnes, 1901)

930139 ***Scotura*** *annulata* (Guérin-Méneville, 1844)

**930370.1 *Lophocampa bicolor* Walker, 1855**

**930496.1 *Zanclognatha dentata* Wagner & McCabe, 2011**

930582 *Hypena* ***degesalis*** Walker, 1859

930613 *Gonodonta sicheas* (Cramer, **[**1777**]**)

930615 *Gonodonta pyrgo* (Cramer, **[**1777**]**)

930617 *Gonodonta nutrix* (**Stoll, [1780]**)

930627 *Hypocala andremona* (**Stoll, [1781]**)

930633 *Goniapteryx servia* (**Stoll, [1780]**)

**930638.5 *Rhosologia porrecta* Walker, 1865**

930641 *Gabara stygialis* (Smith, 1903)

**syn. *Gabara infumata* (Hampson, 1926)**

930758 *Thysania zenobia* (Cramer, **[**1777**]**)

930792 *Catocala ilia* (Cramer, **[1775]**)

930822 *Catocala californiensis* Brower, 1976

930845 *Catocala grynea* (Cramer, **[1779]**)

930853 *Catocala* ***clintonii*** Grote, 1864

930864 *Cissusa spadix* (Cramer, **[**1780**]**)

930880 *Melipotis novanda* (Guenée, 1852)

**syn. *Melipotis agrotipennis* (Harvey, 1875)**

930924 *Caenurgina erechtea* (Cramer, **[**1780**]**)

930951 *Argyrostrotis flavistriaria* (Hübner, **[**1831**]**)

**syn. *Argyrostrotis herbicola* (Guenée, 1852)**

**syn. *Argyrostrotis contempta* (Guenée, 1852)**

**syn. *Argyrostrotis diffundens* (Walker, 1858)**

**syn. *Argyrostrotis carolina* (Smith, 1905)**

930954 *Argyrostrotis quadrifilaris* (Hübner, **[**1831**]**)

930957 ***Gondysia*** *similis* (Guenée, 1852)

930958 ***Gondysia*** *consobrina* (Guenée, 1852)

**syn. *Gondysia pertorrida* Berio, 1955**

930959 ***Gondysia*** *smithii* (Guenée, 1852)

930960 ***Gondysia*** *telma* (Sullivan, 2010)

930994 *Metria celia* (**Stoll, [1781]**)

931004 *Zaleops umbrina* (Grote, 1883)

**syn. *Zaleops paresa* (Smith, 1906)**

931049 *Zale intenta* (Walker, [1858])

**syn. *Zale woodii* (Grote, 1877)**

931069.1 ***Eulepidotis dominicata* (Guenée, 1852)**

931069.2 ***Eulepidotis persimilis* (Guenée, 1852)**

931084 *Athyrma adjutrix* of authors, not (Cramer, **[**1780**]**)

931086 *Syllectra erycata* (Cramer, **[**1780**]**)

931138 ***Nola involuta* Dyar, 1898**

**syn. *Nola exposita* Dyar, 1898**

**syn. *Nola aphyla* (Hampson, 1900)**

**= *Nola apera* of authors, not Druce, 1897**

931255 *Cydosia nobilitella* (Cramer, **[**1780**]**)

931314 *Ponometia candefacta* (Hübner, **[**1831**]**)

931329 *Ponometia fasciatella* (Grote, **1875**)

*93*1392 *Spragueia margana*(Fabricius, 1794)

**syn. *Spragueia cuviana* (Fabricius, 1798)**

931426 *Acronicta cyanescens* **Hampson, 1909**

931427 *Acronicta vulpina* (**Grote, 1883**)

**931434.1 *Acronicta parallela* (Grote, 1879)**

**931434.2 *Acronicta mansueta* (Smith, 1897)**

931450 *Acronicta theodora* **of authors**, not Schaus, 1894

931467 *Acronicta increta* Morrison, **1874**

931611 *Oxycnemis advena* Grote, 1882

**syn. *Oxycnemis gustis* Smith, 1907**

931617 *Oxycnemis grandimacula* Barnes & McDunnough, 1910

**syn. *Oxycnemis erratica* Barnes & McDunnough, 1913**

**931636.1 *Aleptina arenaria* Metzler & Forbes, 2011**

**931663.1 *Plagiomimicus pyralina* (Schaus, 1904)**

**931725.1 *Azenia procida* (Druce, 1889)**

**syn. *Azenia nepotica* (Dyar, 1912)**

931993 *Condica mersa* (Morrison, 1875)

**syn. *Condica lunata* (Barnes & McDunnough, 1916)**

**931994.1 *Condica luxuriosa* (Dyar, 1926)**

931997 *Condica concisa* (Walker, 1856**)**

**syn. *Condica proxima* (Morrison, 1876)**

932006 *Condica* ***chardra*****(Schaus, 1906)**

**syn. *Condica revellata* (Barnes & Benjamin, 1924)**

932011 *Condica egestis* (Smith, 1894**)**

**syn. *Condica ignota* (Barnes & Benjamin, 1924)**

932014 *Condica cupentia* (Cramer, **[1779]**)

**932087.1 *Schinia carrizoensis* Osborne, 2010**

**932135.1 *Schinia psamathea* Pogue, 2010**

**932154.1 *Schinia poguei* Metzler & Forbes, 2011**

932222 *Spodoptera androgea* (Stoll, **[1780]**)

932223 *Spodoptera eridania* (**Stoll, [1781]**)

**932239.5 *Pseudomarimatha flava* Ferris & Lafontaine, 2010**

**932249.50** *Chytonix palliatricula* (Guenée, 1852)

**932249.51** *Chytonix sensilis* Grote, 1881

**syn. *Chytonix ruperti* Franclemont, 1941**

932328 *Apamea* ***tahoeensis*** Mikkola & Lafontaine, 2009

932709 *Ufeus satyricus* Grote, 1873

ssp. *Ufeus satyricus* ***sagittarius*** Grote, 1883

**syn. *Ufeus electra* Smith, 1908**

**932711** *Ufeus* ***hulstii*** Smith, 1908

932819 *Trichocosmia inornata* Grote, 1883

**syn. *Trichocosmia drasteroides* (Smith, 1903)**

**932832 *Anarta*** *inconcinna*(Smith, [1888])

**syn. *Anarta castrae* (Barnes & McDunnough, 1912)**

**syn. *Anarta ultra* (Barnes & Benjamin, 1924)**

**syn. *Anarta montanica* (McDunnough, 1930)**

932836 *Anarta obesula* (Smith, 1904)

**syn. *Anarta subalbida* (Barnes & Benjamin, 1924)**

932847 *Scotogramma submarina* (Grote, 1883)

**syn. *Scotogramma addenda* Barnes & Benjamin, 1924**

932849 *Scotogramma densa* Smith, 1893

**syn. *Scotogramma megaera* Smith, 1899**

932880 *Lacanobia radix* (Walker, **[**1857**]**)

**syn. *Lacanobia desperata* (Smith, 1891)**

932909 *Hecatera dysodea* ([Denis & Schiffermüller], 1775)

933009 *Lasionycta sierra* Crabo & Lafontaine, **2009**

933017 *Lacinipolia lustralis* (Grote, 1875)

**syn. *Lacinipolia selama* (Strecker, 1898)**

933062 *Lacinipolia comis* (Grote, 1876)

**syn. *Lacinipolia lunolacta* (Smith, 1903)**

**933093.1 *Homorthod*es** *euxoiformis* (Barnes & McDunnough, 1913)

933105 *Protorthodes incincta* (Morrison, 1874)

**syn. *Protorthodes smithii* (Dyar, 1904)**

933121 *Ulolonche fasciata* Smith, [1888]

**syn. *Ulolonche marloffi* (Barnes & Benjamin, 1924)**

933128 *Pseudorthodes vecors* (Guenée, 1852)

**syn. *Pseudorthodes imora* (Strecker, 1898)**

**syn. *Pseudorthodes calceolaris* (Strecker, 1900)**

933143 “*Orthodes*” *noverca* (Grote, 1878)

**syn. “*Orthodes*” *vauorbicularis* (Smith, 1902)**

**syn. “*Orthodes*” *delecta* (Barnes & McDunnough, 1916)**

**933156.1** “*Hexorthodes*” *accurata* (H. Edwards, 1882)

933169 *Neleucania praegracilis* (Grote, 1877)

**syn. *Neleucania bicolorata* (Grote, 1881)**

**syn. *Neleucania niveicosta* Smith, 1902**

**syn. *Neleucania citronella* Smith, 1902**

**syn. *Neleucania suavis* (Barnes & McDunnough, 1912)**

933210 *Xanthopastis* ***regnatrix***** (Grote, 1863)**

**syn. *Xanthopastis timais* of authors, not Cramer, [1780])**

**933210.1 *Xanthopastis moctezuma* Dyar, 1913**

933280 *Copablepharon spiritum* Crabo & Fauske, 2004

**ssp. *Copablepharon spiritum spiritum* Crabo & Fauske, 2004**

**ssp. *Copablepharon spiritum lutescens* Crabo & Fauske, 2004**

**ssp. *Copablepharon spiritum bicolor* Crabo & Fauske, 2004**

933282 *Copablepharon canariana* McDunnough, 1932

ssp. *Copablepharon canariana* ***contrasta***McDunnough, 1932

933308 *Euxoa adumbrata* (Eversmann, 1842)

ssp. *Euxoa adumbrata* ***thanatologia*** (Dyar, 1904)

933596 *Xestia speciosa* (Hübner, [1813])

ssp. *Xestia speciosa* ***apropitia*** (Benjamin, 1933)

## Notes

**p. 2 & p. 14 Subfamily Lymantriinae Hampson, Tribe Nygmiini Holloway, 1999** – This tribe should be added to the check list for the Old World genus *Euproctis* Hübner, [1819]. *Euproctis* was listed in error in the tribe Leucomini.

**p. 3 & p. 39 Subfamily Eulepidotinae** – The year of publication is 1895, not 1985.

**p. 3 & p. 39 Tribe Eulepidotini** – The year of publication is 1895, not 1985.

**p. 4 & p. 64 Subfamily Agaristinae** – The author and year are Boisduval, 1833, not Herrich-Schäffer, [1858].

**930067 *Macrurocampa marthesia*** – The year of publication was deduced through external sources, so it should be shown in brackets. See note under 930617.

**930125 *Elasmia packardii* –** New combination from Metzler and Knudson (2011).

**930126 *Elasmia cave* –** This species was previously misidentified in North America as *Hippia insularis*, a species described from Cuba (Metzler and Knudson 2011).

**930126.1 *Elasmia mandela* ssp. *santaana* –** Populations of *Elasmia mandela* occurring in south-central United States are segregated as a separate subspecies from the nominate form in Central America (Metzler and Knudson 2011).

**930130 *Symmerista suavis* –** The species name was misspelled as *sauvis* in both Franclemont and Todd (1983) and in Lafontaine and Schmidt (2010).

**930139 *Scotura annulata* –** This new generic combination was created when Miller (2009) synonymized the genus *Zunacetha* Walker, 1863 with *Scotura* Walker, 1854.

**930370.1 *Lophocampa bicolor* –** The presence of this species in southern Texas has previously been overlooked, since it resembles washed-out specimens of *Lophocampa caryae*. It is currently known only from Big Bend NP, Brewster Co., TX (specimens in Canadian National Collection and Texas Lepidoptera Survey Collection).

**930496.1 *Zanclognatha dentata* –** Addition (see Wagner and McCabe 2011).

**930582 *Hypena degesalis***– The species name was misspelled as *degasalis* in Lafontaine and Schmidt (2010) and Franclemont and Todd (1983).

**930613 *Gonodonta sicheas* –** See note under 930617.

**930615 *Gonodonta pyrgo* –** See note under 930617.

**930617 *Gonodonta nutrix* –** The author of this species is Casper Stoll, who finished the last part of Pieter Cramer’s work after Cramer’s death, so the names are credited to Stoll. The title pages of the first two volumes of Pieter Cramer’s work are dated 1779, but were actually published in a series of parts between 1775 and 1777. The last two volumes are dated 1782 but were published between 1779 and 1782 and the title pages were added later when the series was completed. The 34 issues that made up the four volumes were published between 1775 and Cramer’s death in 1776 and posthumously until 1780 by Casper Stoll. During 1780 the series was continued by Stoll and the names of the new species on plates 305 to 400 are credited to him. The dates are mostly deduced from external sources, so these should be shown in brackets. Only volume 4, pages 165 to 252 (plates 373 to 400) was actually published in 1782. The dates of publication of the 34 issues of texts and plates are taken from “AnimalBase – Early Zoological Literature On-line” maintained by the Zoological Institute of the University of Göttingen, Germany at (http://www.animalbase.uni-goettingen.de [accessed September 2011]).

**930627 *Hypocala andremona* –** See note under 930617.

**930633 *Goniapteryx servia* –** See note under 930617.

**930638.5 *Rhosologia porrecta*** – A specimen of this mainly Mexican species from Texas is in USNM with labels “Victoria, TX” “JD Mitchell Coll.” Ed Knudson (pers. comm.) provided additional information: “Joseph Daniel Mitchell was a pioneer biologist in Texas (1848–1922). He lived in Victoria TX from 1891 to about 1920 and was known for collecting insects, mollusks, etc. No doubt the specimen is genuine.”

**930641 *Gabara stygialis* –** *Gabara infumata* (Hampson, 1926), **syn. n.** [formerly # 930642], is a form of *Gabara stygialis* in which the dark streak along the middle of the forewing is greatly reduced.

**930758 *Thysania zenobia* –** See note under 930617.

**930792 *Catocala ilia* –** See note under 930617.

**930822 *Catocala californiensis*** – A nominate subspecies is listed for *Catocala californiensis*, but currently no other subspecies are recognized so the subspecies entry should be deleted.

**930845 *Catocala grynea*** – See note under 930617.

**930853 *Catocala clintonii* –** The correct original spelling is *Catocala clintonii*, not *clintoni*.

**930864 *Cissusa spadix* –** See note under 930617.

**930880 *Melipotis novanda* –** The name *Melipotis agrotipennis* (Harvey, 1875) is a synonym of *Melipotis novanda* (Guenée, 1852), **syn.** **n.**, not a synonym of *Melipotis agrotoides* (Walker, 1858).

**930924 *Caenurgina erechtea* –** See note under 930617.

**930951 *Argyrostrotis flavistriaria* –** The year of publication was deduced through external sources, so it should be shown in brackets. Synonymy with *Argyrostrotis herbicola* [formerly # 930947], *Argyrostrotis contempta* [formerly # 930948], *Argyrostrotis diffundens* [formerly # 930949], and *Argyrostrotis carolina* [formerly # 930950] from Sullivan and Lafontaine 2011.

**930954 *Argyrostrotis quadrifilaris* –** The year of publication was deduced through external sources, so it should be shown in brackets.

**930957 *Gondysia similis* –** Generic combination from Sullivan and LeGrain 2011.

**930958 *Gondysia consobrina* –** Generic combination and synonymy with *Gondysia pertorrida* from Sullivan and LeGrain 2011.

**930959 *Gondysia smithii* –** Generic combination from Sullivan and LeGrain 2011.

**930960 *Gondysia telma* –** Generic combination from Sullivan and LeGrain 2011.

**930994 *Metria celia* –** See note under 930617.

**931004 *Zaleops umbrina* –** The name *Zaleops paresa*, **syn.** **n.** [formerly # 931005], represents a dark form of *Zaleops umbrina* in which the forewing is mainly blackish brown with pale shading in the costal area and along the outer margin. The type specimens in AMNH (*paresa*) and USNM (*umbrina*) were both examined.

**931049 *Zale intenta* –** This revised synonymy, proposed by Schmidt 2010, was omitted from the check list.

**931069.1** ***Eulepidotis dominicata* –** This species was inadvertently omitted from the 2010 check list. It was included in the 1983 MONA list (Franclemont and Todd 1983) on the basis of a report of the specimen from Texas published by Grote (1879) and discussed by Todd (1961). A recent specimen from Mission, Texas, 11 November 2002, by Leroy Koehn is in the TLSRC. A full report of the occurrence of this species and the next (*Eulepidotis persimilis*) is being prepared by Ed Knudson and associates.

**931069.2** ***Eulepidotis persimilis* –** A specimen of thisspecies was collected at Mission, Texas, 17 October 2011, by Mike Rickard. The specimen is deposited in the TLSRC.

**931084 *Athyrma adjutrix* of authors –** See note under 930617.

**931086 *Syllectra erycata* –** See note under 930617.

**931138** ***Nola involuta* –** *Nola involuta* Dyar, 1898, **stat. rev.**, was described from California and occurs from southern California to southern Texas. It is consistently different in wing markings from *Nola apera* Druce, 1897, from Jalapa, Mexico, such as the presence of a black patch along the base of the forewing costa that is absent from *Nola apera*, so we treat *Nola involuta* as a valid species. *Nola exposita* Dyar, 1898, **syn. rev.** [Type locality: Phoenix, Arizona], is revised from the synonymy of *Nola apera* to the synonymy of *Nola involuta*.The holotype of *Nola aphyla* (Hampson, 1900), **syn. rev.** [formerly # 931134], is a badly rubbed specimen of *Nola involuta*; both names are based on southern Californian material.

**931255 *Cydosia nobilitella* –** See note under 930617.

**931314 *Ponometia candefacta* –** The year of publication was deduced through external sources, so it should be shown in brackets.

**931329 *Ponometia fasciatella* –** The year of publication is 1875, not 1975.

**931392 *Spragueia margana* –** The name *Pyralis cuviana* Fabricius, 1798, has until recently been assigned to the Tortricidae as an unrecognized name. The type specimen has now been located in the Zoological Museum, University of Copenhagen, Denmark (ZMUC). *Pyralis cuviana* Fabricius, 1798, **syn. n.**, is a synonym of (and a form of) *Pyralis margana* Fabricius, 1794. The species is currently known as *Spragueia margana* (Fabricius, 1794) and the synonym is *Spragueia cuviana* (Fabricius, 1798), **comb. n.** The type locality was originally given as ‘Habitat Kiliae’ [Kiel in northern Germany], so this should be corrected to “the Americas.” [Contributed by Ole Karsholt and Don Lafontaine].

**931426 *Acronicta cyanescens*** – The author and year are Hampson, 1909, not Guenée, 1852.

**931427 *Acronicta vulpina* –** The author and year are Grote, 1883, not Guenée, 1852.

**931434.1 *Acronicta parallela* –** Nearly identical externally to *Acronicta falcula* and some populations of *Acronicta mansueta*, and all three taxa were treated as conspecific by Lafontaine and Schmidt (2010). Additional study of specimens from key geographic areas, and re-examination of the type material, reveals that *Acronicta parallela*, **stat. rev.**, is a valid species and our previous concepts of the taxa were erroneously based on geographic variants of *Acronicta mansueta*.

**931434.2 *Acronicta mansueta* –** Eastern Great Plains populations are nearly identical externally to *Acronicta parallela*, with which it previously was treated as conspecific by Lafontaine and Schmidt (2010). As noted under 931434.1, *Acronicta mansueta*, **stat. rev.**,is distinct from *Acronicta paralella*.

**931450 *Acronicta* sp nr *theodor*a** – The species recorded in Arizona is an undescribed species related to *Acronicta theodora*. *Acronicta theodora* is known only from Mexico.

**931467 *Acronicta increta* –** The year of publication is 1874, not 1974.

**931611 *Oxycnemis advena* –** *Oxycnemis gustis*, **syn. n.** [formerly # 931614] is a new synonym of *Oxycnemis advena* following Robert Poole (*Noctuidae of North America* – http://www.nearctica.com/moths/noctuid/noctuidae.htm) [accessed September 2011] [Contributed by Robert Poole].

**931617 *Oxycnemis grandimacula* –** *Oxycnemis erratica*, **syn. n.** [formerly # 931616] is a new synonym of *Oxycnemis grandimacula* following Robert Poole (*Noctuidae of North America* – http://www.nearctica.com/moths/noctuid/noctuidae.htm) [accessed September 2011] [Contributed by Robert Poole].

**931636.1 *Aleptina arenaria* –** Addition (see Metzler and Forbes 2011a in references).

**931663.1** ***Plagiomimicus pyralina* –** Specimens of thisspecies have recently been found in Sunnyside Canyon, Huachuca Mountains, Arizona by Bruce Walsh. We tentatively retain the species in *Plagiomimicus* Grote following Poole (1989). It differs from *Plagiomimicus*, and all other Stririini, in having a frontal tubercle with a large central carina projecting out like a shark fin; the vesica has a large, spined, subbasal diverticulum, and the clasper is very long and thin, projecting more than half way across the valve. For these reasons we place the species in the list at the end of *Plagiomimicus*. The identity of the species was confirmed by Robert Poole.

**931725.1 *Azenia procida* –** *Azenia procida* (Druce, 1889), **stat. rev.**, previously was treated as a subspecies of *Azenia edentata* Grote, 1883, by Franclemont and Todd (1983), but differs in wing markings, male genitalia, and the shape of the frontal process. *Azenia edentata* has a clear-yellow forewing with tiny triangular costal dots and the outer margin of the frontal process is wavy, convex centrally and concave near the lateral margins. *Azenia procida* occurs in two color forms, one yellow and the other a dull yellowish brown. It differs from *Azenia edentata* in that the forewing has larger, more diffuse dark spots, the outer margin of the frontal process is evenly convex, and the DNA barcode is more than 4 % different from that of *Azenia edentata*. This taxon previously was treated as a form of *Azenia edentata* ssp. *procida* (Druce, 1889), but is now treated as a synonym of *Azenia procida* (*Azenia nepotica* (Dyar, 1912), **syn. n.**). The name *nepotica* represents the darker yellowish-brown form of *Azenia procida*. The male genitalia, shape of the frontal process, and DNA barcode are like those of the typical form of *Azenia procida*.

**931993 *Condica mersa* –** *Condica lunata*, **syn. n.** [formerly # 932009] is a new junior synonym of *Condica mersa* following Robert Poole (*Noctuidae of North America* – http://www.nearctica.com/moths/noctuid/noctuidae.htm) [accessed September 2011] [Contributed by Robert Poole].

**931994.1 *Condica luxuriosa* –** This mainly Mexican species is reported from Arizona by Robert Poole (*Noctuidae of North America* – http://www.nearctica.com/moths/noctuid/noctuidae.htm [accessed September 2011]). It is related to *Condica albolabes* (Grote, 1880), but the forewing of *Condica luxuriosa*has more black shading and the white speckling is more extensive, especially on the reniform spot and subterminal line. The genitalia of the two species also differ. [Contributed by Robert Poole].

**931997 *Condica concisa* –** *Condica proxima*, **syn. n.** [formerly # 932000], is newly synonymized with *Condica concisa* to follow Robert Poole (*Noctuidae of North America* – http://www.nearctica.com/moths/noctuid/noctuidae.htm [accessed September 2011]). The unique holotype is lost, but the original description best fits that of *Condica concisa*. [Contributed by Robert Poole].

**932006 *Condica chardra* –** *Condica revellata*, **syn. n.** [formerly # 932006] is synonymized with *Condica chardra* following Robert Poole (*Noctuidae of North America* – http://www.nearctica.com/moths/noctuid/noctuidae.htm). [Contributed by Robert Poole].

**932011 *Condica egestis***– The holotype of*Condica ignota*, **syn. n.** [formerly # 932012], in the USNM is an unusually pale specimen of *Condica egestis*. It is not clear if the specimen is naturally pale or is bleached from being exposed to too much light.

**932014 *Condica cupentia* –** See note under 930617.

**932087.1 *Schinia carrizoensis* –** Addition (see Osborne 2010).

**932135.1 *Schinia psamathea* –** Addition (see Pogue 2010).

**932154.1 *Schinia poguei* –** Addition (see Metzler and Forbes 2011b).

**932222 *Spodoptera androgea* –** See note under 930617.

**932223 *Spodoptera eridania* –** See note under 930617.

**932239.5 *Pseudomarimatha flava* –** Inadvertently omitted from Lafontaine and Schmidt 2010. For description, see Ferris and Lafontaine (2010).

**932249.50, 932249.51 *Chytonix* Grote –** This genus belongs in the tribe Elaphriini. It has the divided sacculus and weakened area on the costa of the valve (e.g., see Lafontaine et al. 2010). With the move in position, the numbers of the two species change from 932713 and 932714 to 932249.50 and 932249.51.

**932249.51 *Chytonix ruperti* –** *Chytonix ruperti*, **syn.** **n.** [formerly # 932715],is a synonym of *Chytonix sensilis*. The external characters (blackish in lower medial area, white dot by pm line, blackish basal area, black wedges on inner margin of subterminal line) are all variable and occur in varying combinations throughout. In the genitalia, the width of the tegumen and the form of clasper (a divided process versus a V-shaped sclerite), also are variable and both forms of clasper occur in the *ruperti* form (including the type series) and the typical *sensilis* form. A distinctive Florida form has a different barcode and may be a separate species, but structural characters are variable - just like in *sensilis/ruperti*.

**932328 *Apamea tahoeensis* –** The correct spelling of the specific name is ***tahoeensis*** not *tahoensis*.

**932681 *Andropolia contacta*** – See the correction to Note 506 for this species below.

**932386 “*Oligia*” *divest*a** – This species was returned to its longstanding position in the *Oligia* Hübner group of genera by Lafontaine and Schmidt (2010), without explanation, after having been transferred to the genus *Chytonix* by Franclemont and Todd (1983). Genital and larval characters indicate that “*Oligia*” *divesta* (Grote, 1874) belongs in the tribe Xylenini, subtribe Apameina, as does the *Oligia* group of genera. *Chytonix* Grote, however, belongs in the tribe Elaphriini (see Note 932249.1 above).

**932709 *Ufeus satyricus* –** The correct spelling of the subspecific name is ***sagittarius*** not *saggiatarius*. The lectotype of *Ufeus electra*, **syn.** **n.** [formlerly # 932711], in AMNH is a western specimen (from Oregon) of *Ufeus satyricus*, so the name becomes a synonym of *Ufeus satyricus sagittarius*.

**932711 *Ufeus hulstii* –** The name for the western counterpart of *Ufeus plicatus* becomes *Ufeus hultsii*, **stat.** **rev.**, with the transfer of *Ufeus electra* to the synonymy of *Ufeus satyricus*. The correct spelling for the specific name is *hulstii*, not *hulsti*.

**932819 *Trichocosmia inornata* –** The lectotype of *Trichocosmia drasteroides*, **syn.** **n.** [formerly # 932820], in USNM is a more maculate form of *Trichocosmia inornata*.

**932832 *Anarta inconcinna* –** The holotype of *Anarta inconcinna*, **comb.** **n.** [formerly # 932861], is a senior synonym of *Anarta castrae*, **syn.** **n.** [formerly # 932829], *Anarta ultra*, **syn.** **n.** [formerly a subspecies of *Anarta castrae*], and *Anarta montanica*, **syn.** **n.** [# 932832], The holotype of *Anarta inconcinna* from New Mexico is a form of the species in which the reniform and medial areas are paler than other areas of the forewing. *Anarta ultra* represents the more typical, evenly-colored, orange-brown form of the species. *Anarta montanica* is a more northerly and westerly yellow-brown form. The placement of the species here, rather than in *Scotogramma*, where *inconcinna* has previously been placed, is because the species is most closely related to *Anarta oregonica* and *Anarta alta*. The barcodes of populations in Alberta and British Columbia differ by less than 0.5% from those from New Mexico, whereas those of *Anarta montanica* differ from those of *Anarta oregonica* and *Anarta alta* by more than 1.5%.

**932836 *Anarta obesula*** – The holotype of*Anarta subalbida*, **syn. n.** [formerly # 932835], from Whitehorse, Yukon,in USNM, represents the same species as the lectotype of *Anarta obesula* from Calgary, Alberta, in AMNH.

**932847 *Scotogramma submarina* –** The holotype of *Scotogramma addenda*, **syn. n.** [formerly # 932852],from Colorado, represents the same species as *Scotogramma submarina* from Montana.

**932849 *Scotogramma densa* –** This species, described from the Argus Mountains of California, is the same species as *Scotogramma megaera*, **syn. n.** [formerly # 932854], described from Glenwood Springs, Colorado. Both lectotypes are in USNM.

**932880 *Lacanobia radix* –** Lafontaine and Schmidt (2010) followed Franclemont and Todd (1983) and Poole (1989) in listing *Mamestra desperata* Smith, 1891 [formerly # 932775], as a species of *Orthosia* Ochsenheimer. We had overlooked the synonymy of *Mamestra desperata* with *Lacanobia radix* by McCabe (1980), but follow it here. We have examined the lectotype of *Mamestra desperata* in USNM and agree with the synonymy by McCabe.

**932909 *Hecatera dysodea* –** Note 551 on the discovery of this Eurasian species in North America should be corrected as follows. The first collections of *Hecatera dysodea*in Oregon were made in 2003 at Dufur (Wasco Co.) [not in 2005 at The Dalles]. The species was first collected in Washington in 2008 at Stevenson (Skamania Co.). Additional vouchers are in ODA (Richard Worth, pers. comm.).

**933009 *Lasionycta sierra* –** The year of publication is 2009, not 2010.

**933017 *Lacinipolia lustralis*** – The holotype of*Lacinipolia selama*, **syn. n.** [formerly # 933034], in theFMNH is a typical specimen of *Lacinipolia lustralis*. The type locality of *Lacinipolia selama* is nominally Dallas, Texas, but the species has never been collected there, so the holotype could be mislabelled as to locality.

**933062 *Lacinipolia comis*** – The holotype of*Lacinipolia lunolacta*, **syn. n.** [formerly # 933064],in theBMNH is a dark female of *Lacinipolia comis*.

**933093.1 *Homorthodes euxoiformis*** – This species [formerly # 933153] is transferred from the genus *Hexorthodes* McDunnough to the genus *Homorthodes* McDunnough as *Homorthodes euxoiformis*, **comb. n.** The genital characters and barcode results suggest a close relationship to *Homorthodes dubia* (Barnes & McDunnough, 1912).

**933105 *Protorthodes incincta* –** The syntypes of*Protorthodes smithii*, **syn. n.** [formerly # 933106], in USNMrepresent yet another form of the extremely variable species *Protorthodes incincta*.

**933121 *Ulolonche fasciata*** – The holotype of*Ulolonche marloffi*, **syn. n.** [formerly # 933122],represents the same species as *Ulolonche fasciata*. Both holotypes are in USNM and both types are from New Mexico.

**933128 *Pseudorthodes vecors*** – The holotype of*Orthodes imora*, **syn. n.** [formerly # 933130], is a pale reddish specimen of *Pseudorthodes vecors* from Wisconsin. The holotype of *Orthodes calceolaris*, **syn. rev.** [formerly # 933129], is a reddish specimen of *Pseudorthodes vecors* with white dusting on the transverse lines. It was described from Long Island, New York, and was raised to species status by Franclemont and Todd (1983), presumably on the basis of the discussion in Forbes (1954) that refers to populations from Long Island and south as having two rather than one generation a year. There is nothing in the genitalia or barcode results to suggest that southern populations are specifically distinct from northern ones. The types *Orthodes imora* and *Orthodes calceolaris*, are in the FMNH.

**933143 “*Orthodes*” *noverca* –** Additional research on this species has confirmed the suggestion in Lafontaine and Schmidt (2010, note 591) that “*Orthodes*” *vauorbicularis*, **syn. n.** [formerly # 933144], and “*O.*” *delecta*, **syn. n.** [formerly # 933145], are geographic forms of a single species. Typical “*Orthodes*” *noverca*,a contrastingly-marked orange-brown form of the Great Basin and Rocky Mountains region, intergrades into the lightly-marked gray-brown forms of the Pacific Northwest (*vauorbicularis*) and California (*delecta*). Also, there are no differences in the genitalia or DNA barcodes to suggest that more than one species is involved.

**933156.1 “*Hexorthodes*” *accurata* –** The species number is changed from 933154 to associate the species with “*Hexorthodes*” *citeria* and “*Hexorthodes*” *emendata*.

**933169 *Neleucania praegracilis* –** Although the holotype of *Neleucania praegracilis* is lost, the details of the original description ― eyes hairy, resembling *Heliophila pallens* [*Mythimna pallens* (Linnaeus, 1758)], but with the forewing pale yellowish white and the slender habitus of *Senta defecta* Grote, 1874 [“*Photedes*” *defecta* (Grote, 1874)] ― leave no doubt as to the identity of this species. The list of synonyms (*Neleucania bicolorata* (Grote, 1881), **syn. n.** [formerly # 933167], *Neleucania niveicosta* Smith, 1902, **syn. n.** [formerly # 933168], *Neleucania citronella* Smith, 1902, **syn. n.**, *Neleucania suavis* (Barnes and McDunnough, 1912), **syn. n.** [formerly # 933165], is due mainly to the variability of the forewing color. It varies from an even pale yellowish white to a dark orange brown; in darker forms the veins are streaked and the costa is white. There is no significant geographical variation in external appearance or genital characters that might suggest that any of the names in synonymy might represent a valid species. The species is unusual in that the male has a dark fuscous hindwing, whereas that of the female is almost white, the reverse of the usual situation in the Noctuidae. The only other species in the genus, *Neleucania patricia* (Grote, 1880), differs significantly in appearance and genitalia from *Neleucania praegracilis* and should probably be moved to another genus.

**933210 *Xanthopastis regnatrix* –** The species known as *Xanthopastis timais* (Cramer, [1780]) is now recognized as a species complex consisting of at least six species. This is based on genital differences found by Tim McCabe (pers. comm.) and larval differences described by Dyar (1913a, 1913b, 1919). The name for the species in eastern United States is *Xanthopastis regnatrix* (Type locality: Pennsylvania). This species name was used for the species by Kimball (1965), Wagner (2005) and Wagner et al. (in press). It is characterized by the black patch of scales on the forewing that completely surround the reniform and orbicular spots, the relatively small process on the inner surface of the right valve, and the larval characters described by Dyar (1913a).

**933210.1 *Xanthopastis moctezuma* –** *Xanthopastis moctezuma*, **stat. rev.**, a mainly Mexican species (Type locality: Mexico),) is characterized by the more broken black patch on the forewing, which forms an irregular series of dots on the outer side of the reniform spot, the much larger process on the inner surface of the right valve, and by the larval characters given by Dyar (1913b). It is known from as far north as Brownsville, Texas, but appears to be replaced by *Xanthopastis regnatrix* elsewhere in Texas. A specimen labeled “New Mexico” belongs to *Xanthopastis moctezuma*, but the occurrence of this species in the Southwest needs further confirmation.

**933280** ***Copablepharon spiritum*** – Insert three subspecies of *Copablepharon spiritum* following Crabo and Fauske 2004.

**933282 *Copablepharon canariana* –** The subspecies name ***contrasta*** was misspelled as *contrasa* in the text and index.

**933308 *Euxoa adumbrata***– Subspecies ***thanatologia*** was misspelled as *thantologia* in the text and index.

**933596 *Xestia speciosa* –** Subspecies ***apropitia*** was misspelled as *apropritia* in the text and index.

**p. 114 Note 11** – The reference to Ferguson (1973) should be corrected to Ferguson (1978). The reference is given below.

**p. 121 Note 75** – The reference to Wagner (2008) should be corrected to Wagner et al. (2008). The reference is given below.

**p. 130 Note 177 –** The reference to Fibiger (2009) should be corrected to Fibiger et al. (2009).

**p. 132 Note 205 –** The reference to Pogue (2009) should be corrected to Pogue (2009a).

**p. 152 Note 497** – This note incorrectly spells ***Lomilysis***, a generic synonym of *Brachylomia*, as *Lomolysis*.

**p. 153 Note 506** – This note incorrectly refers to *Andropoliacontrasta*, a misspelling of *Andropolia* ***contacta***.

**p. 156 Note 555 –** The reference to Pogue (2009) should be corrected to Pogue (2009b).

**p. 160 Note 615 –** The reference to Crabo et al. (2004) should be corrected to Lafontaine et al. (2004) and is given below.

**p. 161 Note 642 –** The reference Lafontaine and Crabo (1997) should be corrected to Crabo and Lafontaine (1997).

**p. 165 Note 715 –** The reference Ferguson (1963) was omitted. It is given below.
